# Integration of multiparametric MRI and clinical indicators to predict response to immune-targeted therapy in patients with advanced hepatocellular carcinoma

**DOI:** 10.3389/fonc.2026.1689963

**Published:** 2026-02-11

**Authors:** Shuai Han, Fan Meng, Li-feng Wang, Peng-rui Gao, Hong-kai Zhang, Jin-rong Qu

**Affiliations:** Department of Radiology, Affiliated Cancer Hospital of Zhengzhou University, Henan Cancer Hospital, Zhengzhou, Henan, China

**Keywords:** hepatocellular carcinoma, immune-targeted therapy, inflammatory indices, nomogram, therapeutic response

## Abstract

**Objective:**

The aim of this investigation is to evaluate the efficacy of a predictive model integrating multiparametric MRI and clinical indicators for forecasting the therapeutic response to immune-targeted therapy in patients with advanced hepatocellular carcinoma (HCC).

**Methods:**

This retrospective analysis included 78 patients with HCC who received immune-targeted therapy between January 2021 and October 2024. Abdominal MRI scans were conducted within 2 weeks prior to treatment initiation and again at 8 weeks post-treatment. Complete pre-treatment laboratory data were available for all patients. Based on the Modified Response Evaluation Criteria in Solid Tumors (mRECIST), the patients were categorized into either a disease control group (*n* = 32) or a progression group (*n* = 46). The most discriminative features were selected via LASSO regression, and the optimal predictive factors were constructed based on the λ.1se criterion determined through 10-fold cross-validation. Subsequently, independent predictors were identified using multivariate logistic regression analysis. Prediction models based on imaging, clinical, and combined variables were constructed and evaluated using receiver operating characteristic (ROC) curves. In addition, decision curve analysis and calibration curves were employed to assess the predictive accuracy and discriminative ability of the nomogram. Progression-free survival (PFS) was estimated with Kaplan–Meier analysis.

**Results:**

Independent predictors for response to therapy in advanced HCC included the post-treatment T2 signal intensity ratio (T2 SIR) (*p* = 0.003), post-treatment apparent diffusion coefficient (ADC) mean value (*p* = 0.004), and neutrophil to lymphocyte ratio (NLR) (*p* = 0.013). The areas under the ROC curves for the imaging, clinical, and combined nomogram models were 0.751 (95% CI: 0.639–0.863), 0.614 (95% CI: 0.482–0.744), and 0.811 (95% CI: 0.713–0.910), respectively. Moreover, patients in the high-risk group experienced a significantly shorter median PFS compared to those in the low-risk group (5.0 vs. 7.0 months; *p* < 0.05).

**Conclusion:**

The MRI–clinical nomogram provided effective discrimination of treatment responses to immune-targeted therapy in advanced HCC, thereby enhancing predictive efficiency.

## Introduction

Hepatocellular carcinoma (HCC), the most prevalent primary malignant tumor of the liver, continues to pose significant challenges in clinical diagnosis and management. Epidemiological data indicate that approximately 50% of patients with HCC in China are diagnosed at an intermediate or advanced stage, thereby missing the window for curative surgical intervention (1–3). As a result, systemic therapy becomes the primary treatment option for this patient population. Although considerable progress has been achieved in the diagnosis and management of HCC, overall patient prognosis remains unsatisfactory. The IMbrave150 trial marked a significant milestone by introducing the combined application of targeted agents and immunotherapy in the treatment of unresectable HCC (4). With the rapid advancement of tumor immunotherapy and molecularly targeted therapies, these approaches have demonstrated promising clinical efficacy across a range of solid malignancies (5–7).

In the context of advanced HCC, several studies have indicated that targeted therapy and immunotherapy have contributed to improved disease control and clinical outcomes (8, 9). Chronic inflammation is recognized as a key contributor to tumorigenesis and cancer progression. Emerging evidence has demonstrated that inflammatory processes are closely associated with tumor development and that inflammation-related indices may serve as prognostic markers for various malignancies (10–13). Patients may experience pseudoprogression during immunotherapy, which is likely caused by local inflammatory responses triggered by immune cell infiltration into the tumor microenvironment (TME) (14). Therefore, detecting changes in the TME during treatment can serve as a novel method to evaluate the early efficacy of immunotherapy. MRI capable of providing multi-parametric information on both morphology and function enables TME assessment through the use of specific MRI contrast agents and imaging sequences. It has become the preferred imaging modality for the diagnosis and comprehensive evaluation of liver tumors and provides high sensitivity and specificity for assessing therapeutic responses to immune-targeted therapy in hepatocellular carcinoma (15–17). A model developed by Zhou et al. demonstrated that the combination of MRI radiomic features and clinical variables could effectively predict tumor response and clinical outcomes (18). To date, few studies have investigated the integration of multiparametric MRI features with inflammatory markers for prognostic evaluation in patients with advanced HCC undergoing immune-targeted therapy. Therefore, the present study aimed to develop a predictive model by combining multiparametric MRI features with clinical and biological indicators to assess treatment efficacy in patients with advanced HCC.

## Materials and methods

### Patients

This retrospective study received approval from the institutional review board, with informed consent. The study was conducted in full accordance with the principles outlined in the Declaration of Helsinki.

The inclusion criteria were as follows: (1) diagnosis of HCC confirmed by pretreatment liver biopsy or clinical diagnosis in accordance with the American Association for the Study of Liver Diseases (AASLD) guidelines (19), (2) diagnosis of advanced HCC confirmed by multidisciplinary team (MDT) assessment, indicating ineligibility for curative surgical resection or local ablative therapies, (3) abdominal MRI performed within 2 weeks prior to treatment initiation and at 8 weeks and 6 months following therapy, (4) presence of at least one measurable intrahepatic lesion as defined by the modified Response Evaluation Criteria in Solid Tumors (mRECIST), (5) receipt of standardized immune-targeted therapy within a 6-month window, and (6) availability of complete clinical laboratory results obtained within 2 weeks prior to treatment.

**Figure 1:**
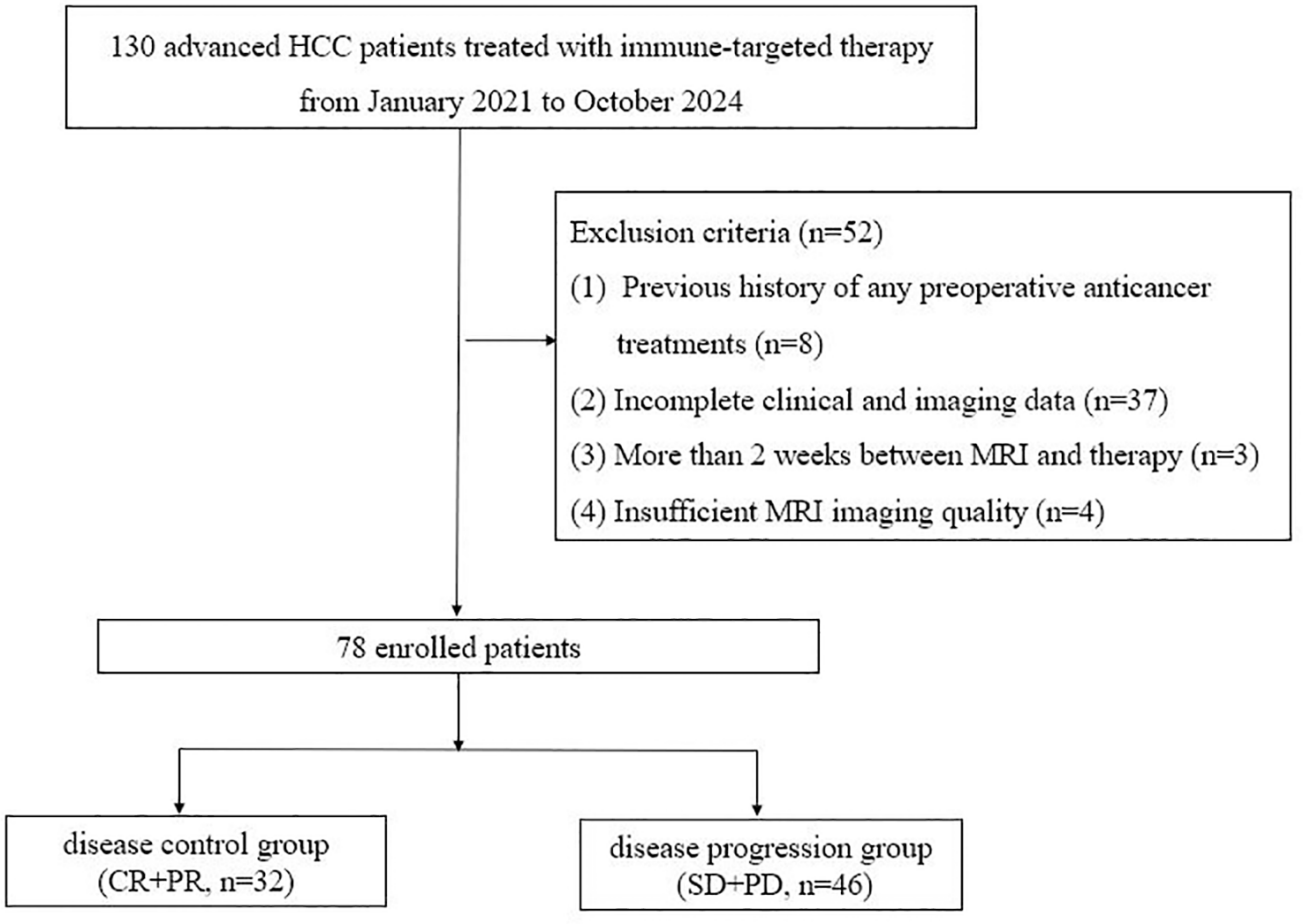
Flowchart of patient selection.

The exclusion criteria were as follows: (1) prior receipt of any preoperative anticancer therapy, (2) incomplete clinical or imaging datasets, (3) an interval exceeding 2 weeks between baseline MRI and therapy initiation, and (4) poor MRI image quality—unsuitable for analysis (see **Figure 1**).

A total of 78 patients met the eligibility criteria, comprising 66 men (84.6%) and 12 women (15.4%), with ages ranging from 32 to 77 years (median age: 56 years). At 6 months following treatment initiation, therapeutic response was assessed based on mRECIST and classified into complete response (CR), partial response (PR), stable disease (SD), and progressive disease (PD). Patients with CR or PR (*n* = 32) were categorized as the disease control group, while those with SD or PD (*n* = 46) comprised the disease progression group (**Table 1**).

**Table 1:** Baseline characteristics across efficacy subgroups.

Characteristic	Total cohort (*n* = 78)	Disease control group (*n* = 32)	Progression group (*n* = 46)
Age, mean ± SD (years)	55.67 ± 8.96	57 ± 15.6	57.5 ± 0.71
Sex, *n* (%)			
Male	66 (84.6%)	30 (93.7%)	36 (78.3%)
Female	12 (15.4%)	2 (6.3%)	10 (21.7%)
Etiology, *n* (%)			
HBV infection	57 (73.1%)	23 (71.9%)	34 (73.9%)
HCV infection	4 (5.1%)	2 (6.3%)	2 (8.7%)
Others (no hepatitis history)	17 (21.8%)	7 (21.9%)	10 (10.9%)
Child–Pugh class, *n* (%)			
A	34 (43.6%)	14 (43.8%)	20 (43.5%)
B	44 (56.4%)	18 (56.2%)	26 (56.5%)
C	0 (0%)	0 (0%)	0 (0%)
Treatment regimens			
Targeted monotherapy	5 (6.3%)	1 (3.1%)	4 (8.7%)
Immune-combined targeted therapy	68 (87.2%)	28 (87.5%)	40 (87.0%)
Immunotherapy monotherapy	5 (6.3%)	3 (9.4%)	2 (4.3%)

Among the 78 patients, five received targeted therapy (sorafenib), 68 received immune-targeted therapy (e.g., sintilimab + lenvatinib), and five received immunotherapy (e.g., tislelizumab) (**Table 2**). The follow-up period ranged from 4 to 38 months (median: 9 months). Progression-free survival (PFS) was defined as the time from treatment initiation to the occurrence of either local tumor progression, distant metastasis, or death attributable to tumor-related causes.

**Table 2:** Baseline characteristics across treatment subgroups.

Characteristic	Total cohort (*n* = 78)	Targeted therapy (*n* = 5)	Immune-targeted therapy (*n* = 68)	Immunotherapy (*n* = 5)
Age, mean ± SD (years)	55.67 ± 8.96	55.5 ± 2.12	62.5 ± 7.78	59.5 ± 9.19
Sex, *n* (%)				
Male	66 (84.6%)	3 (60%)	58 (85.3%)	5 (100%)
Female	12 (15.4%)	2 (40%)	10 (14.7%)	0 (0%)
Etiology, *n* (%)				
HBV infection	57 (73.1%)	5 (100%)	49 (72.1%)	3 (60%)
HCV infection	4 (5.1%)	0 (0%)	4 (5.9%)	0 (0%)
Others (no hepatitis history)	17 (21.8%)	0 (0%)	15 (22.0%)	2 (40%)
Child–Pugh class, *n* (%)				
A	34 (43.6%)	2 (40%)	30 (44.1%)	2 (40%)
B	44 (56.4%)	3 (60%)	38 (55.9%)	3 (60%)
C	0 (0%)	0 ( 0%)	0 (0%)	0 (0%)

### MRI procedure

All MRI scans were performed using a 3.0 Tesla system (MAGNETOM Skyra, Siemens Healthcare, Erlangen, Germany) equipped with an anterior 18-element body coil and a posterior 32-element spine coil array. The patients fasted for 4–6 h prior to imaging and were positioned supine. A gadolinium-based contrast agent (0.1 mmol/kg gadolinium-DTPA) was administered via the antecubital vein at a rate of 2.5 mL/s using an MRI-compatible automated injector pump (Spectris Solaris EP, Medrad, Indianola, PA), followed by an equivalent volume of normal saline. The scanning protocol included the following sequences: fat-suppressed T2-weighted imaging (T2WI-FS), breath-hold T1-weighted in-phase and out-of-phase imaging (T1 in/out phase), diffusion-weighted imaging (DWI), and TWIST-VIBE dynamic contrast-enhanced T1-weighted imaging. Detailed scanning parameters are presented in **Table 3**.

**Table 3:** Detailed scanning parameter settings.

Sequences	T1WI	T2WI (TSE)	DWI	TWIST-VIBE
TR/TE (ms)	3.97/1.29	4,000–8,000/85	3,100/55	3.99/1.25
Slice thickness (mm)	2.5	5.5	5.5	2.5
slice gap (mm)	2.5	6.05	6.05	2.5
Flip angle (°)	12	140	—	10
FOV (mm^2^)	250 × 320	240 × 320	112 × 136	210 × 320
Matrix size	190 × 352	151 × 384	108 × 136	150× 320
*b* value (s/mm^2^)	—	—	50,800	—
NEX	1	1	2	1

### Image analysis

All abdominal plain scan and dynamic contrast-enhanced MRI images were independently analyzed by two experienced radiologists with 15 and 8 years of abdominal MRI diagnostic experience, respectively. If a patient had multiple target lesions, the largest one was selected for subsequent analysis (20). Both radiologists performed the assessment in a blinded manner, with no knowledge of the patients’ clinical data or follow-up outcomes. Any discrepancies in their assessments were resolved through joint discussion with a third senior radiologist who had more than 20 years of experience in abdominal diagnostic imaging to reach a final consensus.

Region of interest (ROI) selection was performed with reference to the aforementioned MRI images, focusing on the solid component of the tumor at the slice corresponding to the maximum tumor diameter. Strict precautions were taken to avoid the tumor margin, large vascular structures, and grossly identifiable cystic and necrotic areas. Three independent ROIs were manually placed at different locations within the solid tumor component, and the apparent diffusion coefficient (ADC) values and T2 signal intensity were measured repeatedly. The average of the three measurements was adopted as the final quantitative result. Meanwhile, a single ROI was selected in the erector spinae muscle region at the same slice to measure the muscle T2 signal intensity, which served as the reference standard. Additionally, the maximum tumor diameter was accurately measured and recorded on the slice with the largest tumor dimension.

The MRI features assessed included tumor location (right lobe vs. other), tumor morphology (irregular vs. round/oval), tumor margin (well-defined vs. ill-defined), enhancement degree (marked vs. moderate/mild), enhancement pattern (homogeneous vs. heterogeneous), DWI target sign (peripheral hyperintensity with central hypointensity on DWI), mosaic architecture (presence of irregular nodules and septa), peritumoral arterial-phase enhancement (transient arterial hyperenhancement surrounding the tumor that becomes isointense on delayed imaging), peripheral halo-like enhancement in venous phase (hyperperfusion of surrounding tissue during the portal venous phase), and tumor capsule status (intact vs. absent/incomplete capsule). Additional MRI features included portal vein thrombosis, arterial-phase rim enhancement, washout appearance, tumor vessels, intratumoral fat, hemorrhage, cystic/necrotic changes, and lymph node metastasis. Quantitative MRI measurements included maximum tumor diameter (pre- and post-treatment), ADC values (ADC_max, ADC_min, ADC_mean), and tumor and muscle T2 signal intensities before and 8 weeks after treatment. Tumor T2 SIR = tumor T2 signal intensity/muscle T2 signal intensity. ADCheterogeneity = (ADCmax – ADCmin)/ADCmean. Post-treatment T2SIR = post-treatment tumor T2 signal intensity/post-treatment muscle T2 signal intensity. Post-treatment ADCheterogeneity = (post-treatment ADCmax – post-treatment ADCmin)/post-treatment ADCmean.

### Clinical data

The collected clinical variables included alkaline phosphatase (ALP), albumin, neutrophils, monocytes, lymphocytes, and albumin to globulin (A/G) ratio. The following inflammatory indices were calculated: NLR = neutrophils/lymphocytes, lymphocyte to monocyte ratio (LMR) = lymphocytes/monocytes, albumin to alkaline phosphatase ratio (AAPR) = albumin/alkaline phosphatase, systemic inflammation response index (SIRI) = (neutrophils × monocytes)/lymphocytes, pan-immune-inflammation value (PIV) = (platelets × neutrophils × monocytes)/lymphocytes, systemic immune-inflammation index (SII) = (platelets × neutrophils)/lymphocytes. The cutoff value for each variable was the value that maximized Youden’s index.

### Statistical analysis

All statistical analyses were performed using SPSS version 20.0 and R version 3.4.1. A two-sided *p*-value <0.05 was considered statistically significant. Interobserver agreement for qualitative and quantitative MRI features was evaluated using kappa statistics and intraclass correlation coefficients (ICCs), respectively. Values of ICC >0.75 and kappa >0.80 were interpreted as indicating good reliability and high consistency. Shapiro–Wilk test was used to assess the normality assumption of the data. Categorical data are presented as frequencies (percentage values), while continuous data with a normal distribution are presented as mean ± standard deviation. Continuous data that do not conform to a normal distribution are presented as median (interquartile range: 25th and 75th percentiles, IQR). Least absolute shrinkage and selection operator (LASSO) regression analysis and univariate logistic regression analysis were employed to identify candidate predictors of treatment efficacy. Variables with *p <*0.1 in univariate analysis were further evaluated using multivariate logistic regression analysis with a stepwise selection method. Three prediction models were constructed: an imaging model, a clinical model, and a combined model. Kruskal–Wallis H test was performed to compare the differences in the expression of the clinical model, imaging model, and combined model across the three treatment groups. Receiver operating characteristic (ROC) curve analysis was used to assess the diagnostic performance of each model and individual predictors, with area under the curve (AUC), sensitivity, specificity, positive predictive value (PPV), and negative predictive value (NPV) calculated. AUC comparisons were performed using DeLong test. A nomogram was developed based on the combined model and evaluated using calibration curves and decision curve analysis (DCA) to assess model accuracy and clinical utility. PFS was estimated using Kaplan–Meier curves, and group comparisons were performed using log-rank test.

## Results

### Identification of independent risk factors for treatment response

**Figure 2:**
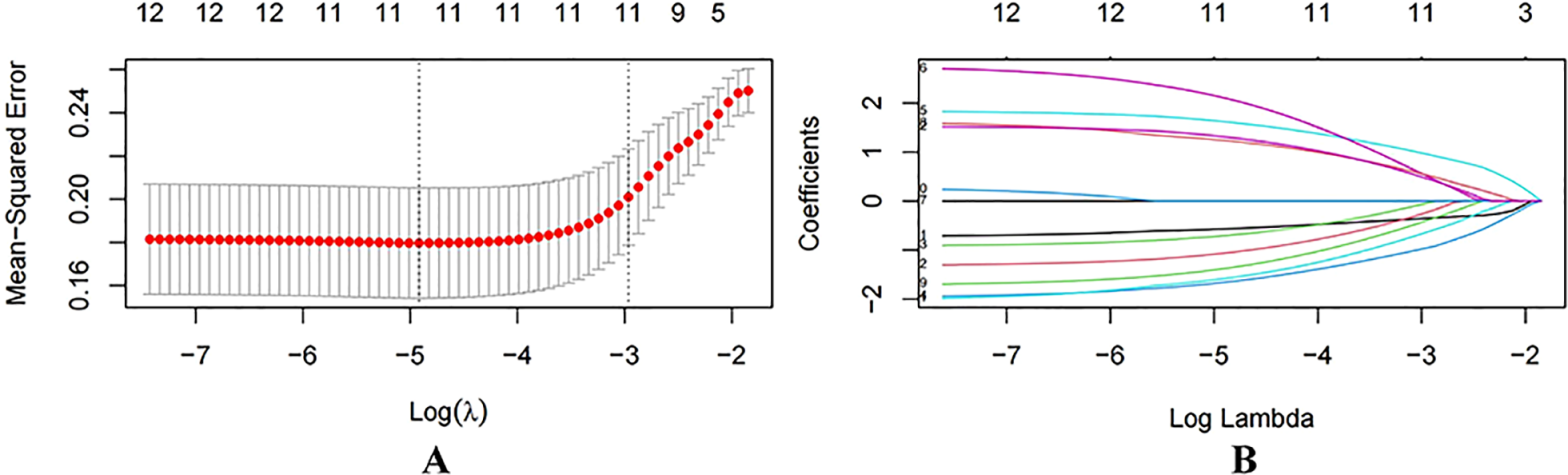
**(A)** LASSO algorithm was used to select the optimal prediction model for HCC efficacy, and the mean square error path of 10-fold cross-validation was adopted. **(B)** LASSO coefficient distribution plot.

**Figure 3:**
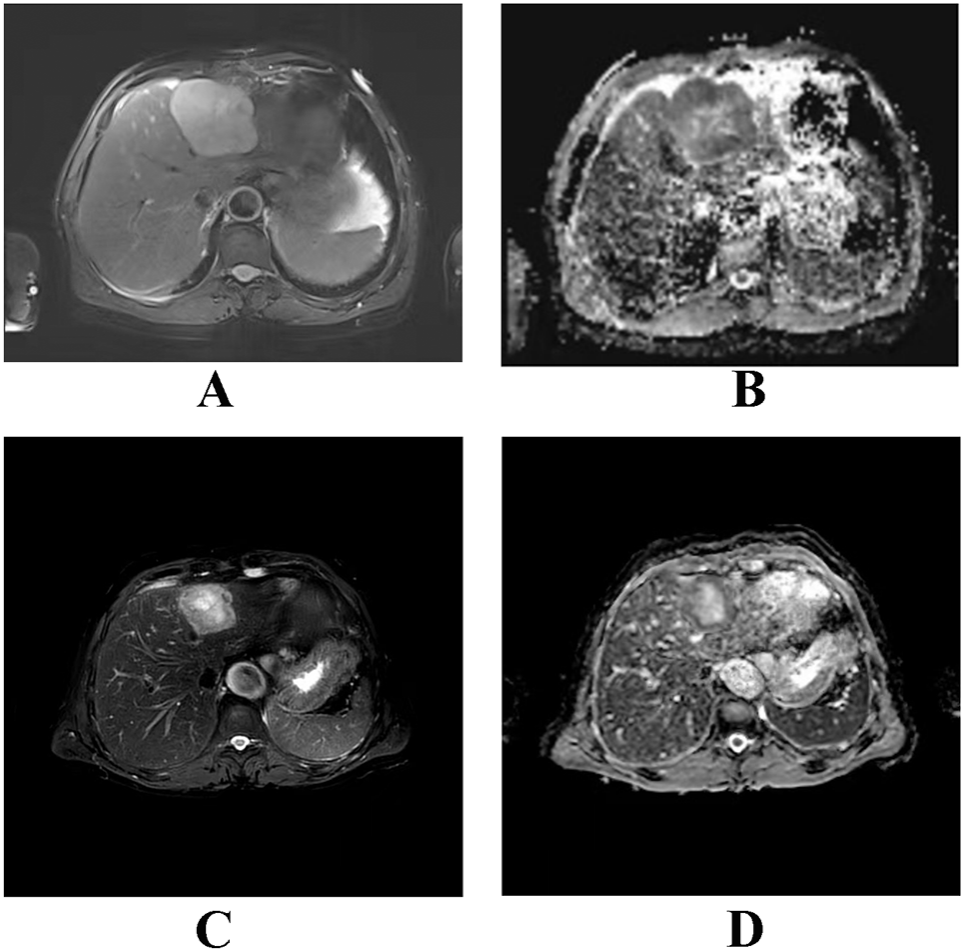
A 62-year-old male patient with HCC in the control group. **(A, B)** The axial T2-weighted image shows a heterogeneous, slightly hyperintense tumor with irregular margins (arrow). T2 SIR was 1.522, and the ADC mean value was 1.075 × 10–^3^ mm^2^/s. **(C, D)** After 2 months of treatment, the tumor shrank significantly (arrow), T2 SIR was 3.833, and the post-treatment ADC mean value was 1.317 × 10–^3^ mm^2^/s. This patient was classified as low risk of progression.

**Figure 4:**
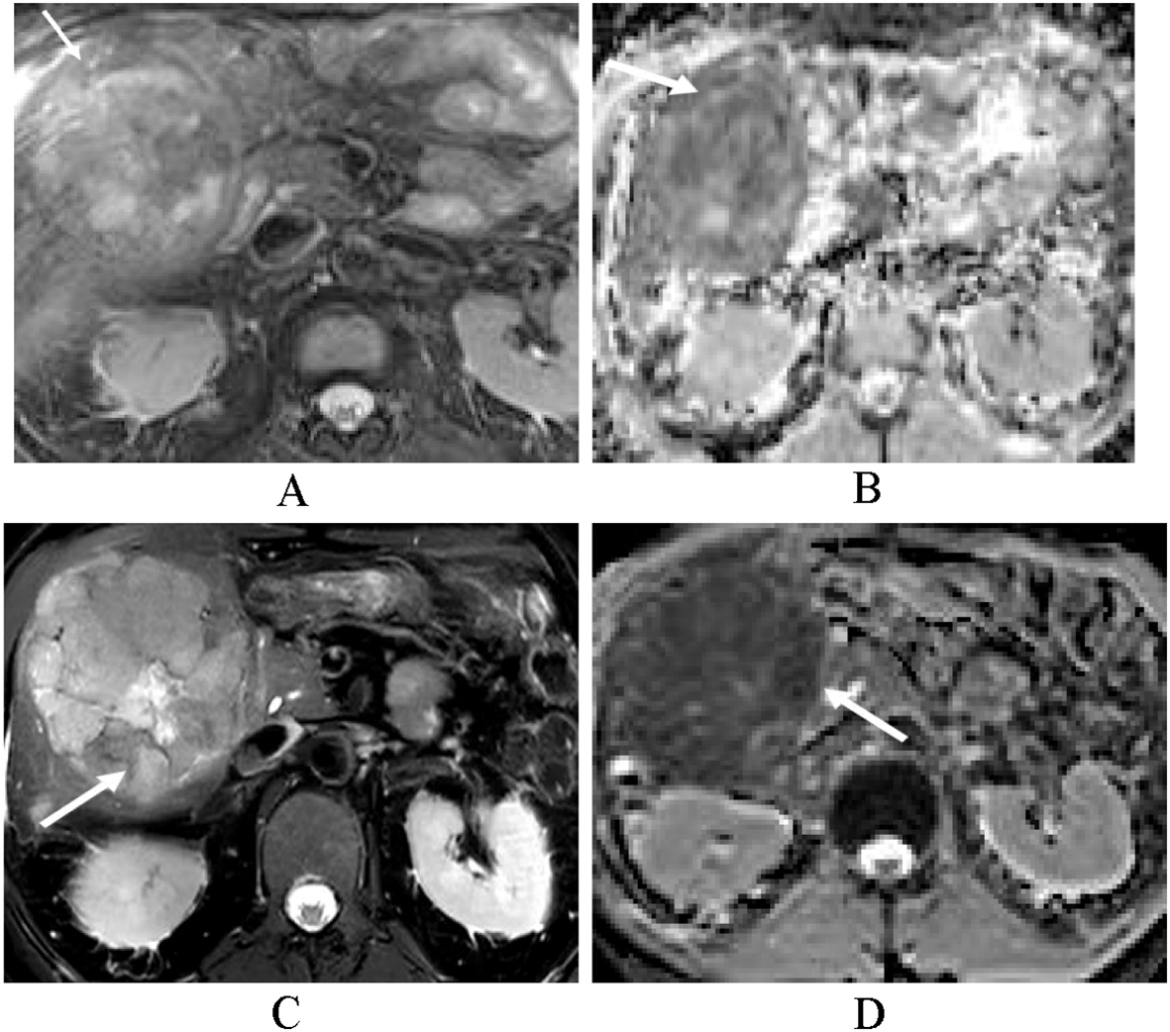
A 59-year-old male patient with HCC in the progression group. **(A, B)** The axial T2-weighted image shows a heterogeneous hyperintense tumor. The ADC map shows a low signal of the tumor (arrow). T2 SIR of the tumor was 2.419, and the ADC mean value was 1.081 × 10–^3^ mm^2^/s. **(C, D)** After 2 months of treatment, the mass increased significantly (arrow), T2 SIR was 2.697, and the post-treatment ADC mean value was 0.968 × 10–^3^ mm^2^/s, and he was predicted to be at a high risk of progression. Finally, this patient progressed at 6 months.

Interobserver consistency analysis demonstrated a good agreement between the two radiologists for both quantitative imaging parameters (ICC = 0.834, *p* < 0.01) and categorical imaging features (kappa = 0.867, *p* < 0.01). Univariate logistic regression analysis and LASSO regression analysis were applied to identify variables associated with treatment efficacy (**Figure 2**). A total of 10 variables with *p <*0.1—including patient sex, post-treatment tumor-to-muscle T2 signal intensity ratio (T2 SIR), post-treatment mean apparent diffusion coefficient (ADCmean), post-treatment maximum ADC (ADCmax), post-treatment maximum tumor diameter, NLR, lymphocyte-to-monocyte ratio (LMR), albumin-to-alkaline phosphatase ratio (AAPR), systemic inflammation response index (SIRI), and systemic immune-inflammation index (SII)—were included in the multivariate logistic regression analysis. The multivariate analysis identified post-treatment T2 SIR (*p* = 0.003), post-treatment ADCmean (*p* = 0.004), and NLR (*p* = 0.013) as independent predictors of therapeutic response in patients with advanced HCC (**Table 4**; **Figures 3**, **4**).

**Table 4:** Baseline characteristics and univariate and multivariate regression risk table.

Characteristic		All patients *N* = 78	Coeff	Univariate analysis		Multifactor analysis	
				OR (95%CI)	P-value	OR (95%CI)	P-value
Sex	Male	66 (84.6)	1.427	4.167 (0.847–20.506)	0.079		
	Female	12 (15.4)	-0.027				
Age (years)		55.67 ± 8.96		0.973 (0.924–1.025)	0.309		
Tumor location	Right lobe	43 (55.1)	0.077	1.080 (0.436–2.678)	0.868		
	Other locations	35 (44.9)					
Tumor morphology	Irregular	51 (65.4)	-0.254	0.775 (0.298–2.020)	0.603		
	Round or oval	27 (34.6)					
Tumor margin	Well-defined	5 (6.4)	-1.083	0.339 (0.036–3.181)	0.343		
	Ill-defined	73 (93.6)					
Enhancement degree	Marked	59 (75.6)	0.232	1.261 (0.434–3.859)	0.670		
	Moderate or mild	19 (24.4)					
Pattern of arterial-phase enhancement	Homogeneous	2 (2.6)	0.373	1.452 (0.087–24.094)	0.795		
	Heterogeneous	76 (97.4)					
Arterial-phase rim enhancement	Present	15 (19.2)	-0.619	0.538 (0.173–1.675)	0.285		
	Absent	63 (80.8)					
Peritumoral arterial-phase enhancement	Present	49 (62.8)	-0.437	0.646 (0.250–1.671)	0.367		
	Absent	29 (37.2)					
Peripheral halo-like enhancement in venous phase	Present	29 (37.2)	-0.532	0.588 (0.230–1.502)	0.267		
	Absent	49 (62.8)					
DWI target sign	Present	13 (16.7)	-0.624	0.536 (0.161–1.778)	0.308		
	Absent	65 (83.3)					
Mosaic architecture	Present	9 (11.5)	-1.196	0.302 (0.070–1.314)	0.110		
	Absent	69 (88.5)					
Portal vein thrombus	Present	39 (50.0)	-0.212	0.809 (0.329–1.997)	0.645		
	Absent	39 (50.0)					
Washout appearance	Present	48 (61.5)	-0.069	0.933 (0.369–2.363)	0.884		
	Absent	30 (38.5)					
Tumor capsule	Intact capsule	20 (25.6)	0.64	1.896 (0.639–5.624)	0.249		
	Absent/incomplete capsule	58 (74.4)					
Tumor vessels	Present	13 (16.7)	0.128	1.137 (0.335–3.858)	0.837		
	Absent	65 (83.3)					
Hemorrhage	Present	38 (48.7)	0.125	1.133 (0.459–2.797)	0.786		
	Absent	40 (51.3)					
Intratumoral fat	Present	8 (10.3)	0.811	2.250 (0.424–11.939)	0.341		
	Absent	70 (89.7)					
Cystic/necrotic changes	Present	36 (46.2)	-0.477	0.621 (0.250–1.541)	0.304		
	Absent	42 (53.8)					
Lymph node metastasis	Present	23 (29.5)	-0.143	0.867 (0.324–2.321)	0.776		
	Absent	55 (70.5)					
T2 SIR	≥1.883	54 (69.2)	-0.547	0.579 (0.204–1.638)	0.303		
	<1.883	24 (30.8)					
Maximum tumor diameter (mm)		95 (59.75, 122.25)	0.747	2.111 (0.783–5.695)	0.140		
Post-treatment T2 SIR	≥1.418	61 (78.2)	1.705	5.500 (1.562–19.362)	0.008	8.692 (2.074–36.429)	0.003
	<1.418	17 (21.8)					
Post-treatment maximum tumor diameter (mm)		71 (48.00, 111.25)	1.876	6.526 (0.774–55.044)	0.085		
ADC value							
ADC max (×10–^3^ mm^2^/s)	≥1.34	34 (43.6)	0.292	1.34 (0.538–3.338)	0.530		
	<1.34	44 (56.4)					
ADC mean (×10–^3^ mm^2^/s)	≥1.09	31 (39.7)	-0.117	1.124 (0.450–2.807)	0.802		
	<1.09	47 (60.3)					
ADC min (×10–^3^ mm^2^/s)	≥0.776	30 (38.5)	-0.601	0.548 (0.217–1.388)	0.205		
	<0.776	48 (61.5)					
ADC heterogeneity	≥0.503	55 (70.5)	0.647	1.909 (0.713–5.115)	0.198		
	<0.503	23 (29.5)					
Post-treatment ADC max (×10–^3^ mm^2^/s)	≥1.107	56 (71.8)	-0.960	0.383 (0.123–1.192)	0.098		
	<1.107	22 (28.2)					
Post-treatment ADC mean (×10–^3^ mm^2^/s)	≥1.085	29 (37.2)	-1.293	0.275 (0.105–0.717)	0.008	0.189 (0.061–0.586)	0.004
	<1.085	49 (62.8)					
Post-treatment ADC min (×10–^3^ mm^2^/s)	≥0.864	21 (26.9)	-0.369	0.691 (0.252–1.896)	0.473		
	<0.864	57 (73.1)					
Post-treatment ADC heterogeneity	≥1.264	10 (12.8)	1.294	3.649 (0.732–18.182)	0.114		
	<1.264	68 (87.2)					
Clinical indicators							
AFP		92.9 (11.23, 1,210)	0.111	1.000 (1.000–1.000)	0.513		
ALP		137 (108.50, 193.25)	0.659	1.933 (0.773–34.836)	0.159		
Albumin-to-globulin (A/G) ratio		1.3 (1.00, 1.60)	-1.083	0.339 (0.036–3.181)	0.343		
Neutrophils		3.92 (2.74, 5.32)	-0.126	0.882 (0.736–1.050)	0.173		
Monocytes		0.34 (0.24, 0.49)	-1.576	0.207 (0.022–1.933)	0.167		
Lymphocytes		1.34 (1.03, 1.63)	0.403	1.496 (0.573–3.906)	0.410		
NLR	≥4.41	14 (17.9)	-1.563	0.210 (0.059–0.210)	0.016	0.162 (0.038–0.682)	0.013
	<4.41	64 (82.1)					
LMR	≥3.24	49 (62.8)	1.167	3.211 (1.234–8.358)	0.017		
	<3.24	29 (37.2)					
AAPR	≥0.33	29 (37.2)	-0.827	0.438 (0.172–1.115)	0.083		
	<0.33	49 (62.8)					
SIRI	≥1.13	32 (41.0)	-1.008	0.365 (0.144–0.925)	0.034		
	<1.13	46 (59.0)					
PIV	≥406.12	14 (17.9)	-0.779	0.459 (0.151–1.397)	0.170		
	<406.12	64 (82.1)					
SII	≥1,096.19	9 (11.5)	-1.564	0.031 (0.051–0.864)	0.032		
	<1,096.19	69 (88.5)					

### Model construction and evaluation

**Figure 5:**
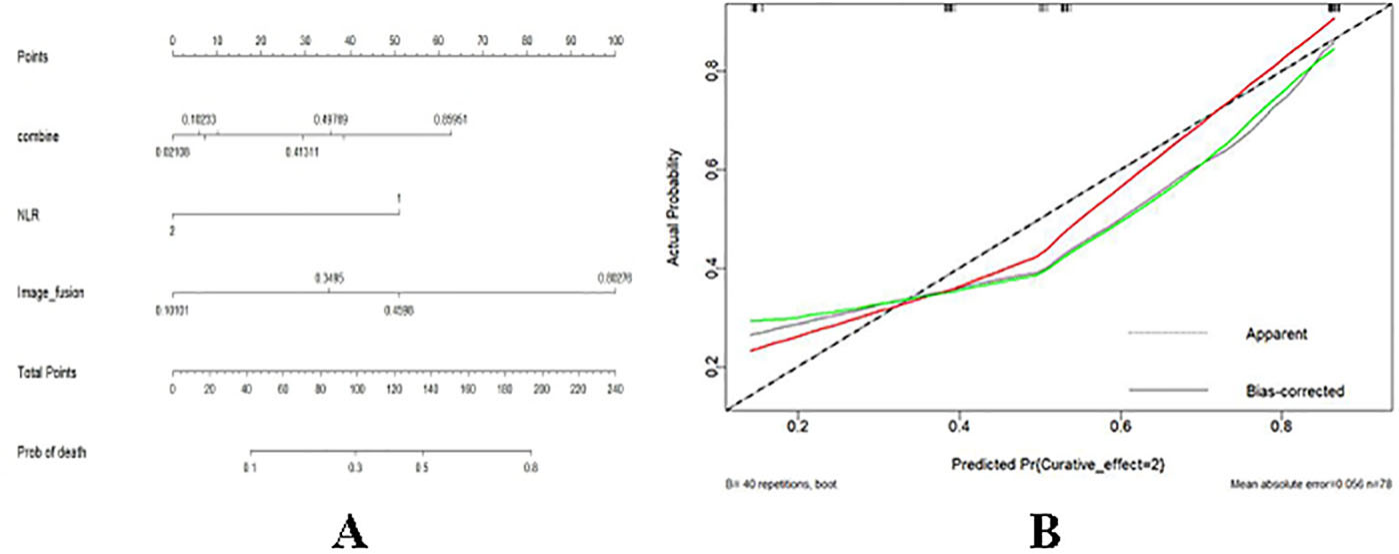
**(A)** Nomogram for predicting therapeutic response probabilities. **(B)** Calibration curve for the nomogram model. On the x-axis is the predicted probability, and on the y-axis is the actual probability of HCC response, and the calibration curve shows a good agreement with the predicted probability and the actual treatment response status.

**Figure 6:**
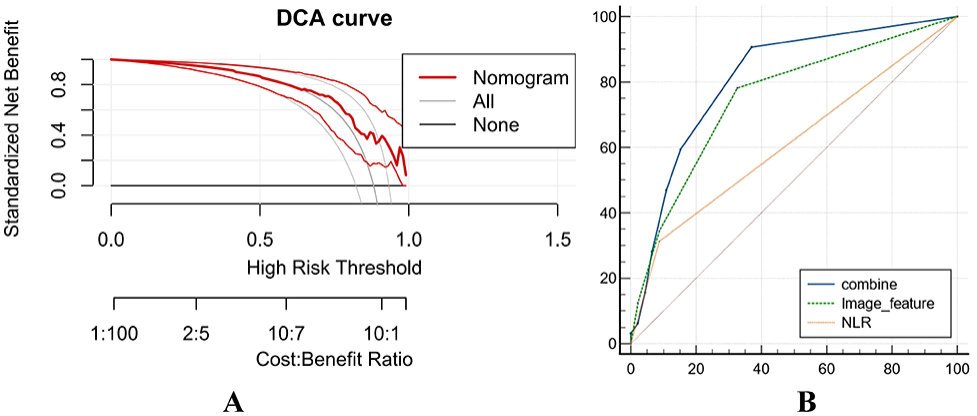
DCA curve of the nomogram model. **(A)** Nomogram is the best decision to predict the maximum net benefit from HCC efficacy compared to other models. **(B)** Receiver operating characteristic (ROC) curves for predicting the efficacy of HCC.

A combined imaging model was developed using post-treatment T2 SIR and post-treatment ADCmean as predictive imaging features. ROC analysis indicated that the combined imaging model had improved diagnostic performance (AUC = 0.751; 95% CI: 0.639–0.863) compared with individual predictors (T2 SIR: AUC = 0.628, 95% CI: 0.512–0.735; ADCmean: AUC = 0.651, 95% CI: 0.534–0.755). DeLong’s test confirmed that the combined model outperformed T2 SIR alone (*Z* = 3.003, *p* = 0.0027). Subsequently, a nomogram was constructed by integrating the imaging features (post-treatment T2 SIR and ADCmean) with NLR. Comparative ROC analysis demonstrated AUCs of 0.751 (95% CI: 0.639–0.863) for the imaging model, 0.614 (95% CI: 0.482–0.744) for the clinical model, and 0.811 (95% CI: 0.713–0.910) for the nomogram model. DeLong’s test demonstrated that the nomogram model was significantly superior to the clinical model (*Z* = 4.023, *p* = 0.0001) (**Table 5**). The calibration curves demonstrated a good agreement between predicted and observed treatment responses, while DCA demonstrated favorable clinical utility of the nomogram (**Figures 5**, **6**).

**Table 5:** Diagnostic efficiency of the three predictive models.

Prediction model	AUC	95% CI	Sensitivity (%)	Specificity (%)	Positive predictive value (PPV)	Negative predictive value (NPV)
Imaging model	0.751	0.641 - 0.842	67.4	78.1	81.58	62.474
Clinical model	0.614	0.496 - 0.721	31.3	91.1	83.501	47.957
Nomogram model	0.811	0.707 - 0.891	63.0	90.6	90.605	62.985

### Comparison among different treatment groups

The AUC values of the targeted therapy group (*n* = 5), immunotherapy group (*n* = 5), and immune-targeted therapy group (*n* = 68) were 0.750 (95% CI: 0.260–1.000), 1.000 (95% CI: 1.000–1.000), and 0.796 (95% CI: 0.705–0.887), respectively. DeLong test showed no statistically significant differences in AUC among the three groups (*p* = 1.000, 1.000, and 1.000, respectively).

### PFS analysis in advanced HCC

**Figure 7:**
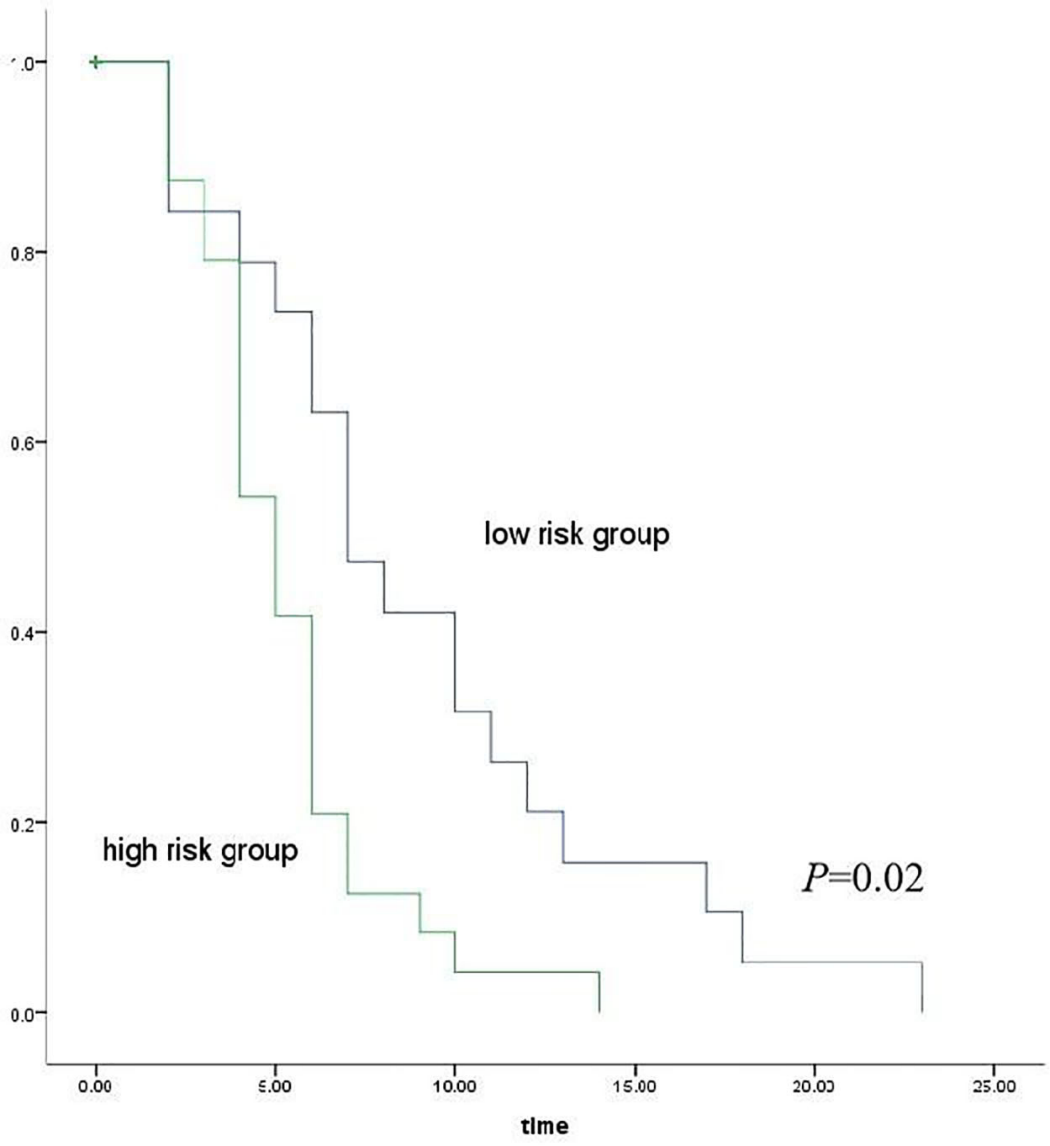
Kaplan–Meier curves illustrate the PFS of patients stratified into high-risk and low-risk groups, with statistical significance assessed using the log-rank test.

PFS was assessed using Kaplan–Meier analysis based on risk stratification derived from the combined nomogram model. The patients were divided into high-risk (score ≥ 0.698) and low-risk (score < 0.698) groups according to the optimal cutoff value determined by the ROC curve. The high-risk group had a significantly shorter median progression-free survival (PFS) than the low-risk group (5.0 months vs. 7.0 months), with statistical significance confirmed by the log-rank test (*p* = 0.02) (**Figure 7**).

## Discussion

This study aimed to develop and validate a nomogram that integrates mpMRI features with the inflammatory biomarker NLR to predict response to immune-targeted therapy in patients with advanced HCC. The core biological rationale rests on a “local–systemic” immune-response axis: effective immunotherapy locally induces immune-cell-driven inflammatory edema, reflected by an elevated T2 SIR, and immune-mediated tumor cell killing with structural disruption, reflected by an increased ADC. Systemically, treatment success shifts the immune-inflammatory balance toward systemic immune activation (lymphocyte expansion) and attenuation of tumor-promoting inflammation (relative neutrophil reduction), together lowering the NLR.

Both mpMRI-derived parameters and NLR were independent predictors of treatment response, and their integration yielded significantly better predictive performance than any single-parameter model. The resulting nomogram showed robust diagnostic accuracy, reliable risk stratification, good calibration, and clinical applicability, thereby offering a practical tool for identifying patients likely to benefit from therapy and supporting individualized treatment decisions.

The liver is a highly immunotolerant organ with unique immunomodulatory functions: it can not only mount efficient immune responses against hepatotropic pathogens but also maintain local and systemic immune tolerance to both self- and foreign antigens (21). This balance is achieved through the coordinated efforts of various conventional antigen-presenting cells (such as dendritic cells, B cells, and Kupffer cells) and non-conventional antigen-presenting cells (such as liver sinusoidal endothelial cells, hepatic stellate cells, and hepatocytes) via both classical and non-classical antigen presentation mechanisms. Consequently, the distinctive anatomical structure and cellular composition of the liver profoundly shape its immunological characteristics (21). On the other hand, the core of tumor immunotargeted therapy is to break the immune suppression of the tumor microenvironment and activate the patient’s own immune effector cells such as T cells to attack tumors. As a result, the focus of efficacy assessment has shifted toward monitoring dynamic changes in the internal composition and immune status of tumors. In this context, developing non-invasive detection methods capable of effectively tracking the dynamic evolution of these key indicators during tumor-targeted immunotherapy is of critical importance. Previous studies have shown that imaging features can reflect the potential molecular and genetic characteristics of tumors (22). Diffusion-weighted magnetic resonance imaging, as a non-invasive functional MRI technique, enables qualitative analysis and quantitative assessment of tumors by quantifying the Brownian motion of water molecules within tissues (23). In this study, patients with hepatocellular carcinoma exhibited an increasing trend in ADC values after receiving targeted immunotherapy compared to baseline; further prognostic analysis confirmed that elevated post-treatment ADC values are an independent protective factor for prognosis in hepatocellular carcinoma patients undergoing targeted immunotherapy. The underlying mechanism may be that when targeted immunotherapy takes effect, activated CD8^+^ cytotoxic T cells infiltrate the tumor tissue and induce tumor cell death through the release of perforin and granzyme or activation of the Fas/FasL pathway—a process immunologically similar to the lymphocyte-mediated “piecemeal necrosis” of hepatocytes seen in autoimmune hepatitis (24). Following treatment, extensive apoptosis and clearance of tumor cells lead to decreased tissue cellularity, widening of the extracellular space, and reduced restriction of water molecule diffusion. This pathophysiological change is specifically reflected in diffusion-weighted imaging as an increase in ADC values. Therefore, the rise in ADC values indirectly reflects the tumor killing efficiency of CD8^+^ T cells and serves as an important imaging biomarker for immunogenic cell death and structural disruption of tumors induced by immunotherapy.

Changes in the T2 SIR are closely associated with alterations in tissue water content (proton density) and cellular composition. In this study, T2 SIR was identified as an independent predictor of prognosis in HCC patients undergoing targeted immunotherapy. The increase in T2 SIR may reflect tissue remodeling triggered by pathological processes such as apoptosis: chemokines released by apoptotic cells recruit immune cells (e.g., microglia, neutrophils, macrophages, and lymphocytes) to migrate toward the lesion area, leading to changes in local cellular composition and the proton microenvironment, which subsequently influence the manifestation of the T2 signal (25, 26). In the context of tumor immunotherapy, an elevated T2 SIR in patients who respond to treatment is not only a manifestation of necrosis and edema after tumor cell death but also indicates the successful infiltration of a large number of immune cells into the tumor tissue, triggering local inflammation and edema. The mechanism is that, after immune-targeted therapy relieves T-cell suppression, antigen-presenting cells (APCs) effectively capture, process, and present tumor antigens—a process similar to the mechanism in autoimmune hepatitis (AIH), where liver APCs present self-antigens leading to effector cell accumulation. This, in turn, recruits cytotoxic T lymphocytes (CTLs) and helper T cells to infiltrate the core area of the tumor. These immune cells and the cytokines they secrete, such as IFN-γ and TNF-α, increase local vascular permeability and interstitial fluid, forming “immune edema.” This edema is a sign of the immune system actively attacking the tumor rather than a mere pathological damage. Therefore, changes in the T2 signal can reflect both compositional alterations associated with apoptosis and serve as an imaging biomarker to monitor the intensity of the local inflammatory response to immunotherapy. In the early stages of treatment, this response may manifest as “pseudoprogression”—where an apparent increase in lesion size on imaging is actually due to immune activation-related edema and inflammation rather than true tumor progression. Hence, when the T2 signal intensity ratio is elevated, it is important to be vigilant about local excessive inflammation or pseudoprogression, and it can be evaluated comprehensively in conjunction with changes in the ADC value.

Studies have confirmed that the combination of T2-weighted imaging features and ADC features can improve the diagnostic accuracy of other types of cancers, such as bladder cancer and prostate cancer (27, 28), which is consistent with the results of this study. This study found that compared with a single parameter (T2 SIR): AUC = 0.628; ADCmean value: AUC = 0.651), the combination of T2 SIR and ADCmean value had better predictive performance (AUC = 0.751). From the perspective of pathophysiology, changes in T2 signal intensity are associated with changes in tumor structure, while ADC values can quantify cell density. Therefore, the combined application of T2 SIR and ADC values can not only accurately distinguish between pseudoprogression and true progression of tumors through the pattern of “elevated T2 SIR + elevated ADC” but also dynamically monitor the biological changes within the tumor during treatment, providing clinicians with earlier and more accurate efficacy evaluation evidence beyond traditional imaging.

Systemic inflammation is tightly correlated with tumor progression and prognosis (27). NLR has been extensively investigated and is identified to be associated with unfavorable clinical outcomes in multiple malignancies, such as lung cancer and colorectal cancer. Notably, elevated NLR in HCC correlates with reduced survival rates (29–31), which aligns with the findings of the present study. Our results demonstrate that NLR acts as a protective factor for advanced HCC patients undergoing immune-targeted therapy, implying that lower NLR correlates with a decreased risk of disease progression or mortality. On one hand, successful immunotherapy activates antigen-specific T cells and promotes their clonal expansion, resulting in an elevated absolute count of peripheral blood lymphocytes. These increased lymphocytes can specifically recognize tumor antigens, activate cytotoxic T lymphocytes, and initiate targeted anti-tumor immune responses to inhibit tumor progression—marking the effective “mobilization” of the body’s immune system. On the other hand, high levels of neutrophils, especially certain subtypes, exert immunosuppressive effects analogous to myeloid-derived suppressor cells. They drive tumor progression through multiple mechanisms, including enhancing tumor cell survival and invasiveness, facilitating metastasis, mediating immunosuppression, remodeling the extracellular matrix, and stimulating angiogenesis (22, 32). Moreover, activated neutrophils form neutrophil extracellular traps, which exacerbate chronic liver disease-related inflammation. They also metabolically reprogram naïve CD4+ T cells, increasing the number of immunosuppressive regulatory T cells and further impairing the body’s anti-tumor immune response (33). Notably, effective immunotherapy can suppress the production of these immunosuppressive cells or induce their apoptosis by modulating the systemic cytokine milieu—such as increasing IFN-γ levels and reducing pro-inflammatory factor release. Simultaneously, the immune balance restored by immunotherapy indirectly leads to a relative decrease in neutrophil counts through regulating systemic inflammatory networks. Thus, a reduction in the NLR not only signifies the effective activation of the systemic immune system but also reflects the attenuation of the body’s inflammatory and immunosuppressive status. This favorable systemic environment provides crucial support for localized anti-tumor immune attacks, with such therapeutic effects observable through imaging signals. The addition of NLR to the imaging model enhanced its overall predictive capacity. The nomogram model achieved an AUC of 0.811, outperforming both the imaging-only model (AUC = 0.751) and the clinical model (AUC = 0.614). PPV and NPV also improved (PPV = 90.605%, NPV = 62.985%) compared to the imaging model (PPV = 81.58%, NPV = 62.474%) and the clinical model (PPV = 83.501%, NPV = 47.957%). These results indicated that the nomogram exhibited a stronger predictive performance. The calibration curves indicated a good agreement between predicted and observed values, while DCA supported its clinical applicability. Furthermore, Kaplan–Meier survival analysis confirmed that patients in the high-risk group experienced significantly shorter PFS than those in the low-risk group. This stratification capacity indicates that the integrated model could assist in identifying patients who may benefit from intensified monitoring or additional therapeutic strategies, potentially improving clinical outcomes. This study employs noninvasive approaches to synchronously quantify immunotherapy-mediated remodeling of the local tumor immune microenvironment (imaging-derived metrics) and systemic immune-inflammatory reprogramming (blood-based biomarkers), thereby enabling an early and accurate assessment of treatment responses.

This study has several limitations that should be acknowledged. First, its retrospective design and single-center setting may introduce selection bias, which could limit the generalizability of the findings. Multicenter prospective studies are necessary to validate the predictive model’s applicability in broader clinical contexts. Second, the relatively small sample size may reduce the statistical power and affect the robustness of the conclusions. Third, external validation in independent and diverse cohorts remains crucial to further confirm the robustness, stability, and clinical applicability of the proposed nomogram. Future research should prioritize addressing these limitations by including larger patient cohorts, adopting prospective multicenter designs, and conducting rigorous external validation to strengthen the evidence base for clinical translation.

## Conclusion

This study successfully developed and validated a nomogram that integrates mpMRI features—including T2 SIR and ADC—with the systemic inflammatory biomarker NLR to predict treatment response in patients with advanced HCC. Based on the “local–systemic” immune-response axis, the model establishes an interpretable link between imaging features and underlying immune mechanisms. The results demonstrate that it not only provides excellent predictive performance and clinical applicability but also offers a non-invasive tool for early response assessment, pseudoprogression differentiation, and personalized treatment guidance.

## Data Availability

The original contributions presented in the study are included in the article/supplementary material. Further inquiries can be directed to the corresponding author.
