# Correction: Integration of multiparametric MRI and clinical indicators to predict response to immune-targeted therapy in patients with advanced hepatocellular carcinoma

**DOI:** 10.3389/fonc.2026.1882353

**Published:** 2026-05-27

**Authors:** Shuai Han, Fan Meng, Li-feng Wang, Peng-rui Gao, Hong-kai Zhang, Jin-rong Qu

**Affiliations:** Department of Radiology, Affiliated Cancer Hospital of Zhengzhou University, Henan Cancer Hospital, Zhengzhou, Henan, China

**Keywords:** hepatocellular carcinoma, immune-targeted therapy, inflammatory indices, nomogram, therapeutic response

The funder “Henan Province Central Plains Talent Program (Nurturing talent Series) (No. RS0011)” was erroneously omitted.

The original version of this article has been updated.

